# Mass Density Measurement of Mineralized Tissue with Grating-Based X-Ray Phase Tomography

**DOI:** 10.1371/journal.pone.0167797

**Published:** 2016-12-21

**Authors:** Regine Gradl, Irene Zanette, Maite Ruiz-Yaniz, Martin Dierolf, Alexander Rack, Paul Zaslansky, Franz Pfeiffer

**Affiliations:** 1 Department of Physics and Institute for Medical Engineering, Technische Universität München, 85748 Garching, Germany; 2 Diamond Light Source, Harwell Science and Innovation Campus, OX11 0DE Didcot, United Kingdom; 3 The European Synchrotron, CS40220, 38043 Grenoble Cedex 9, France; 4 Julius Wolff Institute and Center for Musculoskeletal Surgery, Charité - Universitätsmedizin Berlin, 13353 Berlin, Germany; 5 Institut für Diagnostische und Interventionelle Radiologie, Klinikum Rechts der Isar, Technische Universität München, 81675 München, Germany; Indiana University Purdue University at Indianapolis, UNITED STATES

## Abstract

Establishing the mineral content distribution in highly mineralized tissues, such as bones and teeth, is fundamental in understanding a variety of structural questions ranging from studies of the mechanical properties to improved pathological investigations. However, non-destructive, volumetric and quantitative density measurements of mineralized samples, some of which may extend several mm in size, remain challenging. Here, we demonstrate the potential of grating-based x-ray phase tomography to gain insight into the three-dimensional mass density distribution of tooth tissues in a non-destructive way and with a sensitivity of 85 mg/cm^3^. Density gradients of 13 − 19% over 1 − 2 mm within typical samples are detected, and local variations in density of 0.4 g/cm^3^ on a length scale of 0.1 mm are revealed. This method proves to be an excellent quantitative tool for investigations of subtle differences in mineral content of mineralized tissues that can change following treatment or during ageing and healing.

## Introduction

Highly mineralized tissues, such as teeth and bones, underwent natural selection during millions of years of evolution, hence they are very well adapted to match their specific mechanical-biological function. Moreover, typically they perform better than any implants and are more durable. However, because of their complicated, hierarchical organization, these tissues and the dynamics of mineral content change are far from being completely understood. Bone and dentine in teeth tissues contain a matrix of mineralized collagen fibers. Knowledge about the precise mineral content in these tissues is important for understanding their properties, and is also an indicator for the state of the tissue pathology [1, 2].

Teeth consist of three mineralized tissues known as enamel, dentine, and cementum. The dentine matrix forms the bulk of the tooth and is composed of 45 − 50 vol% (volume percentage) inorganic material (apatite), 30 − 35 vol% organic material (mainly type I collagen) and 4 − 20 vol% fluid (water). It consists of a system of dentinal tubules surrounded by highly mineralized collagen-free peri-tubular dentine and embedded in the intra-tubular dentine. The tubules traverse the structure from the pulp cavity to the region just below the dentine-enamel junction (DEJ). Tubule density, size and orientation vary within the tooth. The diameter of the tubules increases from the DEJ (1 *μ*m) to the pulp chamber (3 *μ*m) and the number of tubules per mm^2^ increases from about 19000 to 75000 [3–5].

Enamel covers the crown of teeth and is the hardest substance in a vertebrate, with hardness values between that of iron and carbon steel [6]. It is dominated by the mineral phase, which is about 95 wt% (percentage by weight). Only about 1 wt% is organic matrix and 4 wt% is water [7]. However, these are only mean values and a variety of values for enamel compositions can be found in literature [8–11].

Often in studies, it is important to precisely quantify the mineral content or mass density of tooth tissues in three dimensions and on relatively large specimens, e.g. to examine differences between healthy and diseased parts of tooth [12–14]. Several methods, like backscattered electron microscopy [15], x-ray ptychography [16] and x-ray absorption microtomography [14] are used for measuring tooth density. However, some of them provide only two-dimensional information or the sample size is limited to a few micrometers or needs destructive sample preparation. For example, backscattered electron microscopy imaging provides quantitative mineral density values with nanometer resolution. However, the approach is limited to those surfaces exposed during the preparation process and does not provide any depth or three dimensional information [15]. Hard x-rays have a higher penetration power than electrons, and different schemes have been proposed to obtain three-dimensional information of mineralized tissues. X-ray absorption microtomography provides volumetric data without the need to slice the sample [9]. However, the sensitivity provided by this method is inherently limited, even when using the highly-brilliant beam available at large-scale synchrotron facilities. Moreover, access to electron density requires a series of assumptions on tissue composition [17]. Phase-contrast x-ray imaging techniques are known to yield higher sensitivity three-dimensional electron density data, down to nanometer resolution [16, 18]. Highly accurate measurement of mineral content in teeth has been demonstrated with x-ray ptychography [16], while magnified x-ray tomography has revealed the ultrastructure of bone with high detail [18]. However, the sample sizes investigated by these methods is often limited to the micrometer range.

Here, we use a technique termed x-ray grating interferometry, previously validated and widely used on a variety of biological tissues (see [19–22]), which allows one to measure quantitatively and accurately the mass density and electron density of different samples (see [19, 23–26]). In this study, teeth samples were selected as an example for one of the most important mineralized biomaterials to demonstrate the potential and application of x-ray grating interferometry in order to discriminate mineralized tissue of different densities. We show that this method, based on phase contrast, allows measurements of the mineral content in three-dimensions on extended samples with an unprecedented spatial sensitivity and a resolution and sample size that allow in-vitro investigation of clinically relevant questions [14].

## Materials and Methods

### Tooth samples

**Fig 1:**
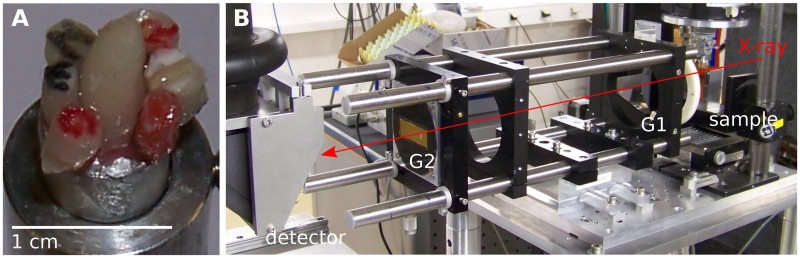
Experimental setup. A) The tooth sections imaged in this study. B) The x-ray grating interferometer at the imaging beamline ID19 of the European Synchrotron Radiation Facility.

Five extracted human teeth (incisor, canine, premolar, 2 × molar) and one pig incisor were used in this study (see Table 1). The pig tooth was obtained from a local butcher, extracted from the discarded jaw-bones after the 4 year old commercial-meat animal was slaughtered. A large warn-down but largely unaffected tooth was selected for this study. All human teeth were obtained from patients undergoing routine dental treatment unrelated to this study, with written informed consent, following an ethics-approved protocol by the Ethical Review Committee of the Charité—Universitätsmedizin Berlin (EA4/002/09). The single samples have a roughly cylindrical shape with diameters varying from 1 to 3 mm and heights varying from 2 to 6 mm, and are glued on an aluminium support for the measurements, see Fig 1A. This sample size was chosen, on the one hand, to provide reasonably large volumes of different regions (enamel / dentin) of several teeth. On the other hand, the size had to be adapted to the experimental constraints, in particular the field of view and the amount of synchrotron beamtime allocated for this experiment.

**Table 1:** Description of the investigated samples.

E	lower anterior (from pig)
G	upper 1st molar, bucco gengival crown region
N	upper canine, palatal aspect, crown-root junction
R	upper canine, incisal enamel and dentine
S	lower premolar, mid buccal crown
U	upper 3rd molar, palatal root tip

### X-ray grating interferometry

The working principles of x-ray grating interferometry (XGI) are described in detail in Refs. [27, 28] and briefly explained below. A standard XGI consists of two gratings: a phase grating (G1) and an absorption analyzer grating (G2). At well-defined propagation distances, called fractional Talbot distances [29], the sinusoidal interference pattern generated by G1 has maximum contrast. The interactions of the x-rays with a sample, usually placed upstream of G1, distort this interference pattern; absorption, phase-shift and small angle x-ray scattering from unresolvable features (dark-field) affect the shape of this pattern independently from each other in different ways. This distorted pattern is compared to a reference pattern recorded without the sample in the beam. In this way, it is possible to retrieve the absorption, differential phase (proportional to the refraction angle), and dark-field images of the investigated object (see Ref. [28, 30]).

**Fig 2:**
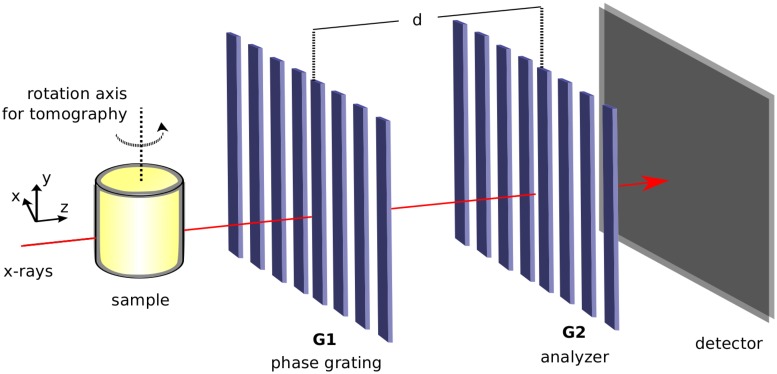
Schematic representation of an x-ray grating interferometer. Usually the sample is placed upstream of the two gratings G1 and G2.

If maintaining a relatively large field of view while optimizing phase sensitivity is desired, the period of the interference pattern generated by G1 often becomes too small to be resolved by the detector. Hence a second grating G2, which has the same period as the interference pattern, is used in the analysis. G2 is placed directly in front of the detector at a fractional Talbot distance *d* from G1. The analysis of the interference pattern is often performed with the phase-stepping technique. A series of images (more than two) is recorded for different transverse positions of G1 (in Fig 2, G1 is moved along the horizontal direction perpendicular to the optical axis) and the desired image signals are extracted with a pixel-wise routine based on Fourier analysis [30].

In a tomographic scan, the sample is rotated around the *y*—axis and hundreds of phase-stepping scans are performed at evenly-spaced viewing angles of the sample. The tomographic reconstruction using absorption and refraction angle projections, usually performed with the filtered-back projection (FBP) algorithm, yields the three-dimensional distribution of the full complexed-value refractive index: *n*(*x*, *y*, *z*) = 1 − *δ*(*x*, *y*, *z*) + ı*β*(*x*, *y*, *z*), where the real part *δ*(*x*, *y*, *z*) is obtained from the phase reconstruction, and the imaginary part *β*(*x*, *y*, *z*), proportional to the linear attenuation coefficient *μ*(*x*, *y*, *z*) = 4*πβ*(*x*, *y*, *z*)/*λ*, where *λ* is the wavelength of the radiation, is computed from the absorption projections [31]. To account for the differential nature of the refraction angle projections, a modified filter in the FBP algorithm is used to obtain the phase tomogram. Moreover, the dark-field volume can also be extracted with a similar procedure from the same dataset [32]. Since the three signals are extracted from the same dataset, the corresponding volumes are inherently registered. In the following, we mainly use the information from the phase and absorption volumes, while the potential of the dark-field signal is discussed in the S1 Appendix.

### From refractive index to mass density

When the measurement is performed with an x-ray energy far from the absorption edges of the materials in the sample, the phase volume gives access to the electron density *ρ*_*e*_(*x*, *y*, *z*) within the investigated specimen [33]:

$$\begin{array}{c} \rho_{e}\left(x,y,z\right)=\frac{2\pi }{r_{0}\lambda^{2}}\delta \left(x,y,z\right)\;, \end{array}$$ (1)

where *r*_0_ is the classical electron radius. The electron density can be transformed to mass density *ρ*_*m*_ [34]

$$\begin{array}{c} \rho_{m}\left(x,y,z\right)=\frac{A\rho_{e}\left(x,y,z\right)}{ZN_{A}} \end{array}$$ (2)

where A is the atomic mass, Z the atomic number and *N*_*A*_ the Avogadro constant. For materials poor in hydrogen such as teeth the approximation *A*/*Z* = 2 g/mol is valid [35]. This gives

$$\begin{array}{c} \rho_{m}\left(x,y,z\right)=\frac{2\rho_{e}\left(x,y,z\right)}{N_{A}}. \end{array}$$ (3)

Thus, by combining Eqs 1 and 3, the mass density is related to the refractive index by the following relation:

$$\begin{array}{c} \rho_{m}\left(x,y,z\right)\left[\frac{g}{{\text{cm}}^{3}}\right]≃\frac{7.48\times 10^{-16}}{\lambda {\left[m\right]}^{2}}\delta \left(x,y,z\right). \end{array}$$ (4)

### Experimental setup

The experiment was performed at the ID19 beamline of the European Synchrotron Radiation Facility (ESRF), Grenoble, France [36]. Setups for grating-based X-ray phase-contrast tomography are already in use at a couple of facilities (e. g. DESY, PSI, Spring8) and could be built up at any of the available synchrotron facilities or even at laboratory sources.

A picture of the two-grating interferometer located at the ID19 beamline is shown in Fig 1B. A Si (111) double crystal monochromator delivered a monochromatic beam (spectral range Δ*λ*/*λ* ≈ 10^−4^) of an x-ray energy of 53 keV (0.23 Å). In this setup, a nickel phase grating (G1) with a period of 2.4 *μ*m and a height of 35 *μ*m was used in combination with an analyzer grating G2 with a period of 2.4 *μ*m and gold structures with a height of 100 *μ*m. Both gratings were produced by the Karlsruhe Institute for Technology (KIT), Karlsruhe, Germany with x-ray lithography [37]. The inter-grating distance was 37 cm, which corresponds to the 3rd fractional Talbot distance at the working energy. The detector was a scintillator-based, lens-coupled CCD camera, with a 10 *μ*m Gadox (Gd_2_O_2_S) scintillator and an effective pixel size of 7.5 *μ*m.

The average visibility of the sinusoidal phase-stepping curve without the sample in the beam can be used to evaluate the performance of the interferometer. It was obtained from a region of 1.5 × 1.5 mm^2^ at the centre of the field of view, and the visibility in one pixel was defined as *V* = (*I*_max_ − *I*_min_)/(*I*_max_ + *I*_min_), where *I*_min_ is the minimum and *I*_max_ is the maximum intensity value of the phase-stepping curve without the sample. The visibility value of 16% suggests a good performance of the instrument, also considering the relatively high x-ray energy which reduces the absorption in the analyzer grating.

For the tomographic measurement, the specimen was immersed in water to reduce artifacts arising from the strong refraction at the sample-air interface [19]. Furthermore, simulations showed that the influence of phase-wrapping artifacts on the delta values are negligible at the employed energy of 53 keV (data is not explicitly shown here). A series of 800 phase-stepping scans were collected over a sample rotation of 180 degrees at equidistant angles. The phase stepping was performed over one grating period in four evenly-spaced steps. The acquisition time per frame was 2 s.

## Results & Discussion

The three-dimensional distributions of the mass density (from the phase volume) and of the linear attenuation coefficient (from the absorption volume) of all samples are obtained simultaneously with the procedure described above. The measured mass density describes the whole tissue including mineral and organic components inside the biocomposites.

**Fig 3:**
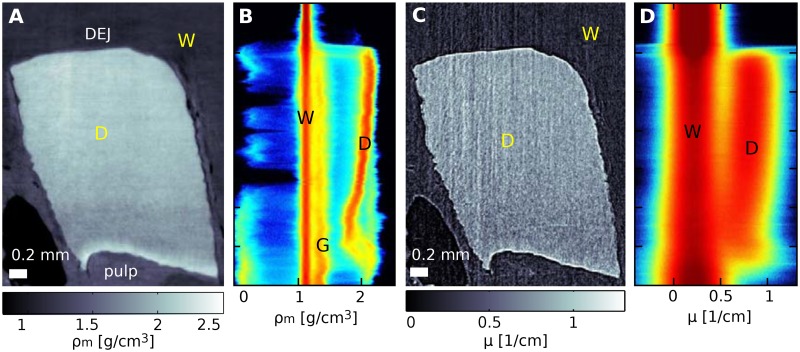
Phase and absorption sagittal slices of sample G and two-dimensional histograms of the entire volumes. Panels A and B show a sagittal slice from the phase volume of sample G and the two-dimensional histogram of the same volume respectively (see S2 Appendix). This data is compared with the corresponding absorption slice (panel C) and absorption histogram (panel D). The vertical axis in the sagittal slices and 2D histograms indicates the height in the volumes.

An example of the same sagittal slice extracted from the phase and absorption data of sample G is shown in Fig 3A and 3C respectively. The top of the sample is taken from a tooth segment near the DEJ, while the lower part of the sample is closer to the pulp. Both absorption and phase signals allow clear separation of the dentine from the surrounding water. However, the visual comparison of these slices immediately highlights the higher quality of the phase image. This is confirmed by the two-dimensional (2D) histogram analysis of the full volumes computed as reported in the S2 Appendix. The 2D histograms are displayed in Fig 3B (phase), and Fig 3D (absorption). The vertical red lines in the 2D histograms are formed by water (straight line at the left used for calibration) and dentine (line at the right). The measured values of about 2.0 g/cm^3^ for mass density and of about 0.8 cm^−1^ for the linear attenuation coefficient are in agreement with previous reports (see [12, 38]). The width of the water peak in the histograms is related to the sensitivity of the measurement. If the data were noise free, all the voxels displaying water would yield the same refractive index value and thus the peak would be infinitely narrow. However, noise and artifacts in the data cause a broadening of the peak. This broadening is more pronounced in the absorption than in the phase data which confirms the higher sensitivity of the latter. Here, the sensitivity of the phase and absorption measurements is estimated to be the full width at half maximum of the water peak, which is 0.38 cm^−1^ for the absorption volume and 85 mg/cm^3^ for the mass density derived from the phase volume. The inclination of the dentine peaks, which can be observed with both contrast modalities, is caused by a refractive index gradient in the axial direction from pulp to DEJ. While this gradient is well defined in the axial direction, the S2 Appendix shows that the properties of the sample do not significantly change in the orthogonal directions. The origin and quantification of the increase in density from pulp to enamel will be further discussed in the following.

### Density gradient

**Fig 4:**
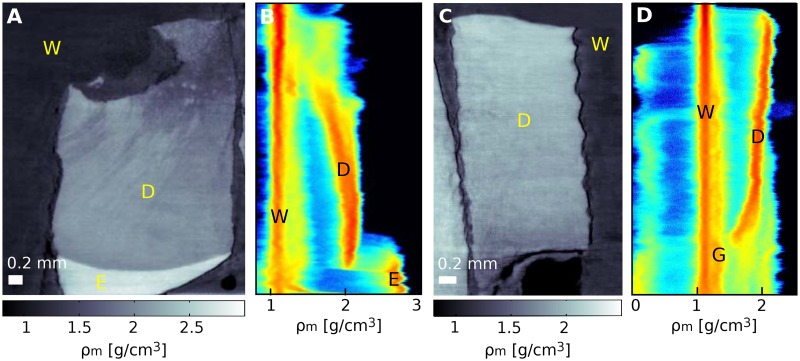
Mass density gradients for sample S and E. In A and C sagittal slices of sample S and E are shown respectively. Panels B and D are the corresponding two-dimensional histograms (see S2 Appendix). The peaks are related to water (W), dentine (D), enamel (E), and glue (G) used to fix the samples. The density variations observed in panel A are probably caused by blind tract formed as a consequence of damage in the dentine.

All examined dentine samples exhibit a well-defined refractive-index gradient in the axial direction. In particular, both the imaginary and real parts of the refractive index (mass density and linear attenuation coefficient) increase from pulp to enamel. Further examples are shown in Fig 4 (samples S and E). This change in density is a known property of dentine and it is explained to be related to a change of both tubule density and mineral content. The tubule density decreases and the mineral content increases from pulp to enamel. Among other techniques, this density variation has previously been quantified using backscattered electron-scanning imaging on chosen slices and with synchrotron radiation absorption-contrast tomography (see [11, 39]).

Here, to evaluate the change in mass density over the examined samples, the densities are calculated for small volumes of interest (VOIs) of 60 × 60 × 10 pixels (450 × 450 × 75 *μ*m^3^) at different heights of the samples. Mass density variations in the range of 13 − 19%, consistent with previous observations, are found over distances of 1 − 2 mm in the investigated samples and summarized in Table 2.

**Table 2:** Mass density variation in dentine.

sample	mass density [g/cm^3^]	distance [mm]
E	1.77 ± 0.02–2.06 ± 0.03	2.0
G	1.79 ± 0.02–2.12 ± 0.03	1.8
N	1.93 ± 0.02–2.10 ± 0.03	1.0
R	1.95 ± 0.02–2.08 ± 0.03	1.0
S	1.83 ± 0.03–2.08 ± 0.04	1.3
U	1.80 ± 0.02–1.99 ± 0.03	1.0

Values are calculated for small VOIs at different heights of the samples. The error takes into account (i) the standard deviation of the gray levels in the VOI and (ii) the reading error in the inter-grating distance estimated to be 0.5 cm. The last column gives the distance over which the density change appears.

### Local density variation

**Fig 5:**
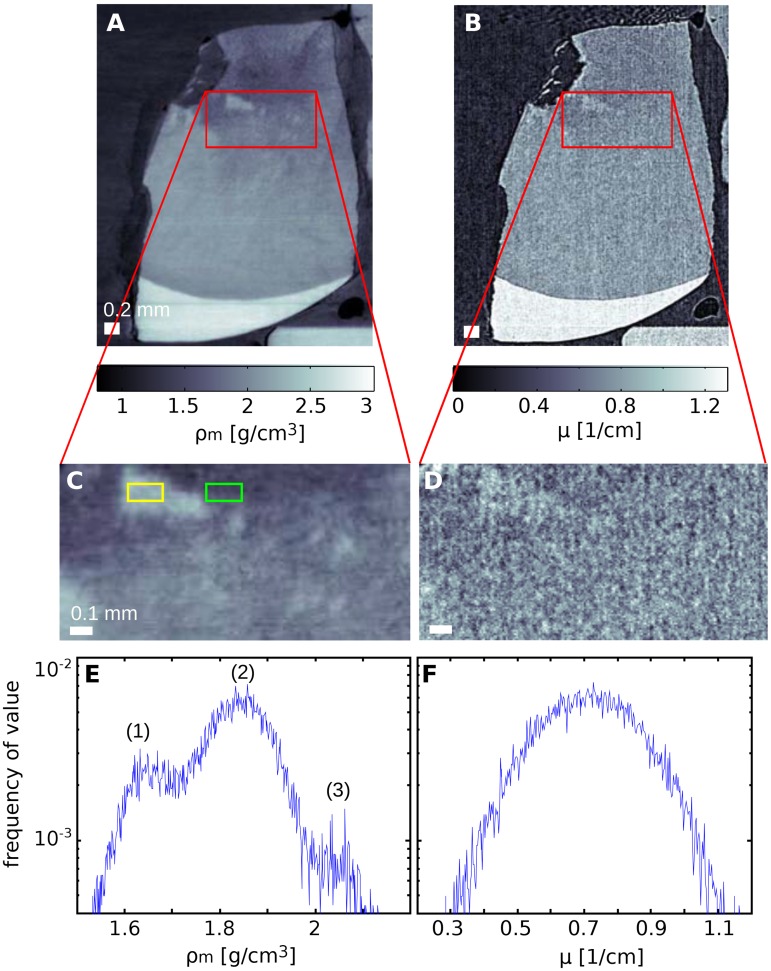
Local density variations detected in dentine. A comparison of phase (A) and absorption (B) sagittal slices of sample S, and enlarged details of these slices (C and D) emphasize the high sensitivity of phase imaging to local density variations. This is confirmed by the histogram analysis of the regions of interest. The phase histogram E features three peaks associated with (1) lower density region at the top-left of panel C, (2) the medium density covering most of the remainder of the same panel, and (3) the high density features. It should be noted that no variation can be detected in the absorption histogram F which consists of just a single peak.

In addition to the gradients discussed above, local mass density variations could be detected based on the high-sensitivity data provided by the phase signal. An example, probably due to to the presence of blind tracts (areas in dentine which are characterized by degenerated odontoblastic processes—dead tracts—and filled with mineral), is shown in Fig 5. Panel A displays a sagittal slice extracted from the phase volume of sample S and Fig 5B the same sagittal slice from the absorption volume. Panels C and D show enlarged details of these slices. While regions with higher density are barely visible in the absorption reconstruction, these areas are clearly revealed in the phase data and indicated with arrows in Fig 5A. The difference in density between high-density regions and their surroundings has been estimated using the regions of interest (ROIs) indicated by yellow and green rectangles in Fig 5C with a size of 75 × 150 *μm*^2^. The values are 2.07±0.01 g/cm^3^ for the higher density region and 1.60±0.01 g/cm^3^ for the outer area, where the errors are determined by the standard deviation within the ROIs. This value yields a contrast-to-noise ratio of 33 computed as proposed in Ref. [19]. The higher sensitivity of the phase signal is also visualized in the histogram analysis shown in Fig 5E and 5F. The histogram of the phase data clearly shows three peaks further described in the caption of the figure, while the absorption data is not sensitive enough to separate such variations in tissue composition.

### Dentine versus enamel

**Fig 6:**
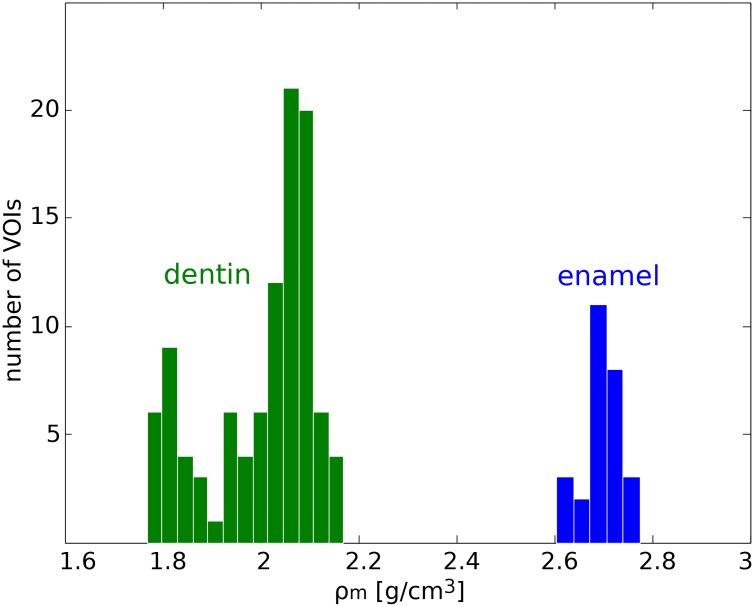
Mass density histogram for dentine and enamel. Mass densities as evaluated in 102 volumes of interest in dentine and 27 volumes of interest in enamel.

Besides dentine, two out of the six samples measured in this study also contained enamel. In the following, the densities of dentine and enamel in our specimens are compared. The mass density values for 102 volumes of interest (VOIs) in dentine (60 × 60 × 10 pixels each) from five human tooth pieces and 27 VOIs in enamel (45 × 45 × 10 pixels each) from two human tooth pieces are computed and displayed in the histogram in Fig 6 [35]. The density values for dentine span 1.79±0.02 g/cm^3^ to 2.12±0.03 g/cm^3^, while the values for enamel span 2.61±0.04 g/cm^3^ to 2.77±0.04 g/cm^3^. The values for dentine are in good agreement with values previously reported: Schwass et al., using a microcomputed tomography system, measured a density of 1.97 g/cm^3^ [40], while Manly et al. using buoyancy experiments with liquids of different densities observed a value of 2.35 g/cm^3^ [38]. Moreover, Manly et al. reported that the root dentine is less dense than the average value of the whole dentine as observed in this study; their average value for the former was 1.94 g/cm^3^ compared to 2.03 g/cm^3^ obtained for the crown region.

The reported mass densities for enamel range from 2.6 g/cm^3^ [38] to 3.0 g/cm^3^ [41]. The average value obtained in this study fits in this range and is in good agreement with the value of 2.72 g/cm^3^ reported by Schwass et al. [40]. The fact that our measurements provide densities in the lower range of the values reported in literature might be due to the fact that we considered VOIs close to the DEJ where the density is known to be lower [41–43].

## Conclusions

We have shown that x-ray grating-based tomography is a valuable tool for three-dimensional quantitative studies on mineralized samples. The reliability of the mass density values obtained with this method is confirmed through comparison with published results obtained with independent techniques.

Compared to other phase-sensitive methods, the object size that can be investigated with x-ray grating interferometry is relatively large and may even extend to a few centimeters. Thus, differently than phase-sensitive nanotomographic methods, entire teeth or bones can be imaged in one scan which requires only a few hours. The mass density values obtained in the reconstructed volume are averaged over the voxel size which may reach tens of micrometers in size.

These characteristics and the ultra-high sensitivity of this technique make it a valuable tool for investigating the density distribution and local density fluctuations in both healthy and pathological bone or tooth specimens. In this manner, x-ray grating-based phase tomography can provide invaluable insight into formation, mineralization dynamics, ageing and pathology of mineralized tissues.

## Supporting Information

S1 Appendix Dark-field information: **Fig A, Dark-field and phase signal of sample G**. Panel A shows a sagittal slice of the dark-field volume of a dentine sample. The scattering signal decreases from the pulp to the surface. Panel B shows the same slice obtained from the phase data.
(PDF)

S2 Appendix Computation of 2D histograms: **Fig A, Construction of the two-dimensional histogram**. Panel A shows the one-dimensional histogram of an axial slice of the reconstructed mass density volume. The peaks are related to water (W), glue (G) and dentine (D). The one-dimensional histograms for each axial slice of the volume are plotted in the two-dimensional histogram in B. The color coding expresses the frequency of values in the volume. **Fig B, Phase volume of sample G and corresponding histograms**. A) Volume rendering of sample G. The analysis of the sample volumes can be performed in axial, sagittal and coronal directions as shown in panel B. The corresponding histograms are in panels C to E.
(PDF)
