# Supplementary material for: Mass Density Measurement of Mineralized Tissue with Grating-Based X-Ray Phase Tomography
Source: PLoS One. 2016 Dec 21;11(12):e0167797. doi: 10.1371/journal.pone.0167797 (PMC5176275; doi:10.1371/journal.pone.0167797)
Supplement: S1 Appendix — Dark-field information: Fig A, Dark-field and phase signal of sample G. Panel A shows a sagittal slice of the dark-field volume of a dentine sample. The scattering signal decreases from the pulp to the surface. Panel B shows the same slice obtained from the phase data. (PDF) [file pone.0167797.s001.pdf]

Supporting information

S1 Appendix.

S1 Appendix. Dark-field information: In addition to the phase and absorption data, grating based tomography (XGI) also provides the dark-field volume. It represents the small-angle x-ray scattering signal from features in the sample that are too small to be directly resolved [1, 2]. The strength of this signal depends on the shape, density and distribution of the scattering features as demonstrated, e.g., by Malecki et al. [3]. From the findings in Ref. [3], we expect that dentinal tubules yield a significant signal with a grating interferometer operating under the conditions of this study. Experimentally, this has been shown in radiography on thin tooth slices [4]. Our data (see Fig. A A) show the first dark-field tomography of dentine tissue, that can be directly compared with the data obtained from the phase reconstructions (see Fig. A B). The signal in the dark-field sagittal slice is stronger at the bottom of the image closer to the pulp, where the tubules are larger in diameter and are denser [5]. This finding confirms, in three-dimensions, the results obtained by Jensen et al. [4]. It should be noted that the region with higher scattering signal also corresponds to the part of the tooth with lower density, probably because a larger volume fraction is empty (occupied by tubules). The fact that a well-defined dark-field signal from the dentinal tubules can be detected in tomography, despite the superposition of the strongly oriented structures, suggests that the extraction of more sophisticated signals such as those measured with x-ray tensor tomography might be possible for these specimens [6].

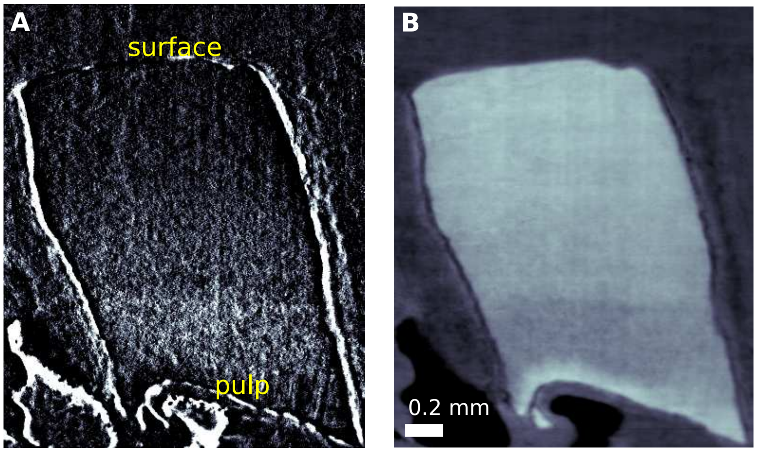

Fig A. Dark-field and phase signal of sample G. Panel A shows a sagittal slice of the dark-field volume of a dentine sample. The scattering signal decreases from the pulp to the surface. Panel B shows the same slice obtained from the phase data.

References

1. Pfeiffer F, Bech M, Bunk O, Kraft P, Eikenberry EF, Brönnimann C, et al. Hard-X-ray dark-field imaging using a grating interferometer. Nature Physics. 2008;7:134–137.

2. Bech M, Bunk O, Donath T, Feidenhans'l R, David C, Pfeiffer F. Quantitative x-ray dark-field computed tomography. Phys Med Biol. 2010;55:5529–5539.

3. Malecki A, Potdevin G, Biernath T, Eggl E, Garcia EG, Baum T, et al. Coherent Superposition in Grating-Based Directional Dark-Field Imaging. PLoS ONE. 2013;8:e61268.

4. Jensen TH, Bech M, Zanette I, Weitkamp T, David C, Deyhle H, et al. Directional x-ray dark-field imaging of strongly ordered systems. Phys Rev B. 2010;82(21):214103.

5. Garberoglio R, Brännström M. Scanning electron microscopic investigation of human dentinal tubules. J Oral Biol. 1976;21:355–362.

6. Malecki A, Potdevin G, Biernath T, Eggl E, Willer K, Lasser T, et al. X-ray tensor tomography. Europhysics Letters. 2014;105:38002.
