# Supplementary material for: Mass Density Measurement of Mineralized Tissue with Grating-Based X-Ray Phase Tomography
Source: PLoS One. 2016 Dec 21;11(12):e0167797. doi: 10.1371/journal.pone.0167797 (PMC5176275; doi:10.1371/journal.pone.0167797)
Supplement: S2 Appendix — Computation of 2D histograms: Fig A, Construction of the two-dimensional histogram. Panel A shows the one-dimensional histogram of an axial slice of the reconstructed mass density volume. The peaks are related to water (W), glue (G) and dentine (D). The one-dimensional histograms for each axial slice of the volume are plotted in the two-dimensional histogram in B. The color coding expresses the frequency of values in the volume. Fig B, Phase volume of sample G and corresponding histograms. A) Volume rendering of sample G. The analysis of the sample volumes can be performed in axial, sagittal and coronal directions as shown in panel B. The corresponding histograms are in panels C to E. (PDF) [file pone.0167797.s002.pdf]

Supporting information

S2 Appendix. Computation of 2D histograms: The quantitative analysis of the sample presented in this study is based on the two-dimensional histogram where each line represents, with an appropriate color coding, a one-dimensional histogram of an axial slice in the volume.

The construction of such a two-dimensional histogram is explained in Fig. A. Panel A of this figure shows a one-dimensional histogram for an axial slice of the reconstructed volume. Three peaks can be identified which correspond to three different materials in the volume: water (W), glue used to fix the sample (G), and dentine (D). This histogram, computed for each axial slice of the volume, can be represented in a single two-dimensional image, as shown in Fig. A B. The horizontal axis of this panel represents the bins of the histogram (as the horizontal axis of the one-dimensional histogram), and the vertical axis indicates the number of the axial slice, which corresponds to the height in the sample. The number of counts in each bin is represented with a color, the corresponding color-bar is shown below the two-dimensional histogram. The values are normalized by the amount of pixels in the axial slices (here: 380×370 pixels). A logarithmic scale is preferred because in this way the histogram peaks can be better visualized. The vertical red line in Fig. A B related to water and marked with the letter "W" is used as calibration as the refractive index of water is known and is constant over the full volume. The theoretical values of $\mu = 0.22$ cm^{-1} and $\delta = 0.80 \times 10^{-7}$ used for this calibration for an x-ray energy of 53 keV are obtained from the Windt and XCOM databases, respectively [1].

The two-dimensional histograms can also be computed along the two orthogonal directions of the volume data (a volume rendering of sample G is shown in Fig. B A), i.e. the sagittal and coronal directions. Slices along these directions are shown in Fig. B B and the respective two-dimensional histograms in Fig. B C to E. The histograms show that the density values do not significantly change in the coronal and sagittal directions (the lines corresponding to dentine in Fig. B (D and E) are straight).

References

1. del Rio MS, Dejus RJ. XOP 2.1 A new version of the x-ray optic software toolkit. AIP Conf Poc. 2004;705:784–787.

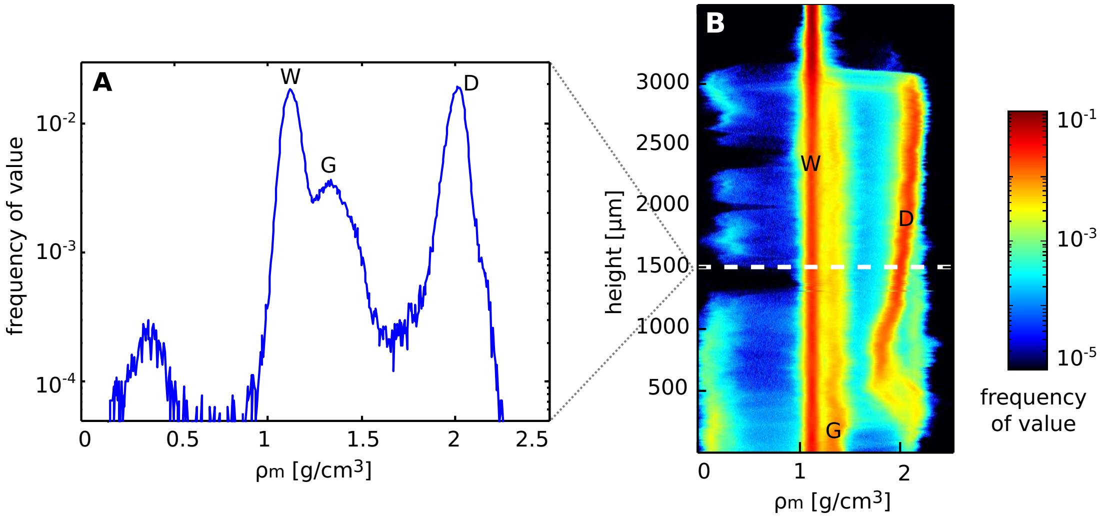

Fig A. Construction of the two-dimensional histogram. Panel A shows the one-dimensional histogram of an axial slice of the reconstructed mass density volume. The peaks are related to water (W), glue (G) and dentine (D). The one-dimensional histograms for each axial slice of the volume are plotted in the two-dimensional histogram in B. The color coding expresses the frequency of values in the volume.

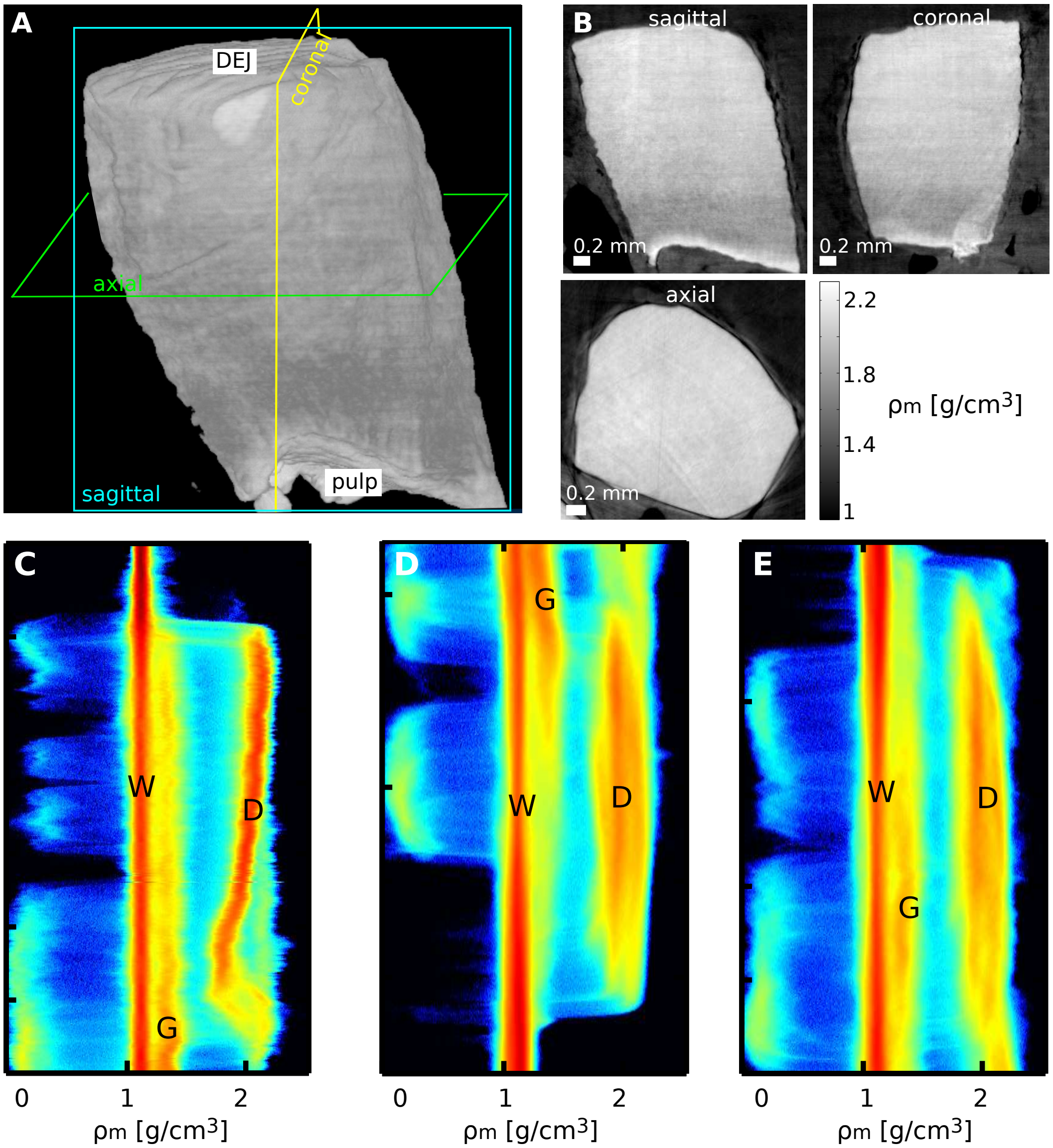

Fig B. Phase volume of sample G and corresponding histograms. A) Volume rendering of sample G. The analysis of the sample volumes can be performed in axial, sagittal and coronal directions as shown in panel B. The corresponding histograms are in panels C to E.
